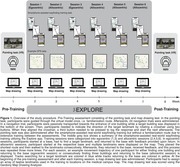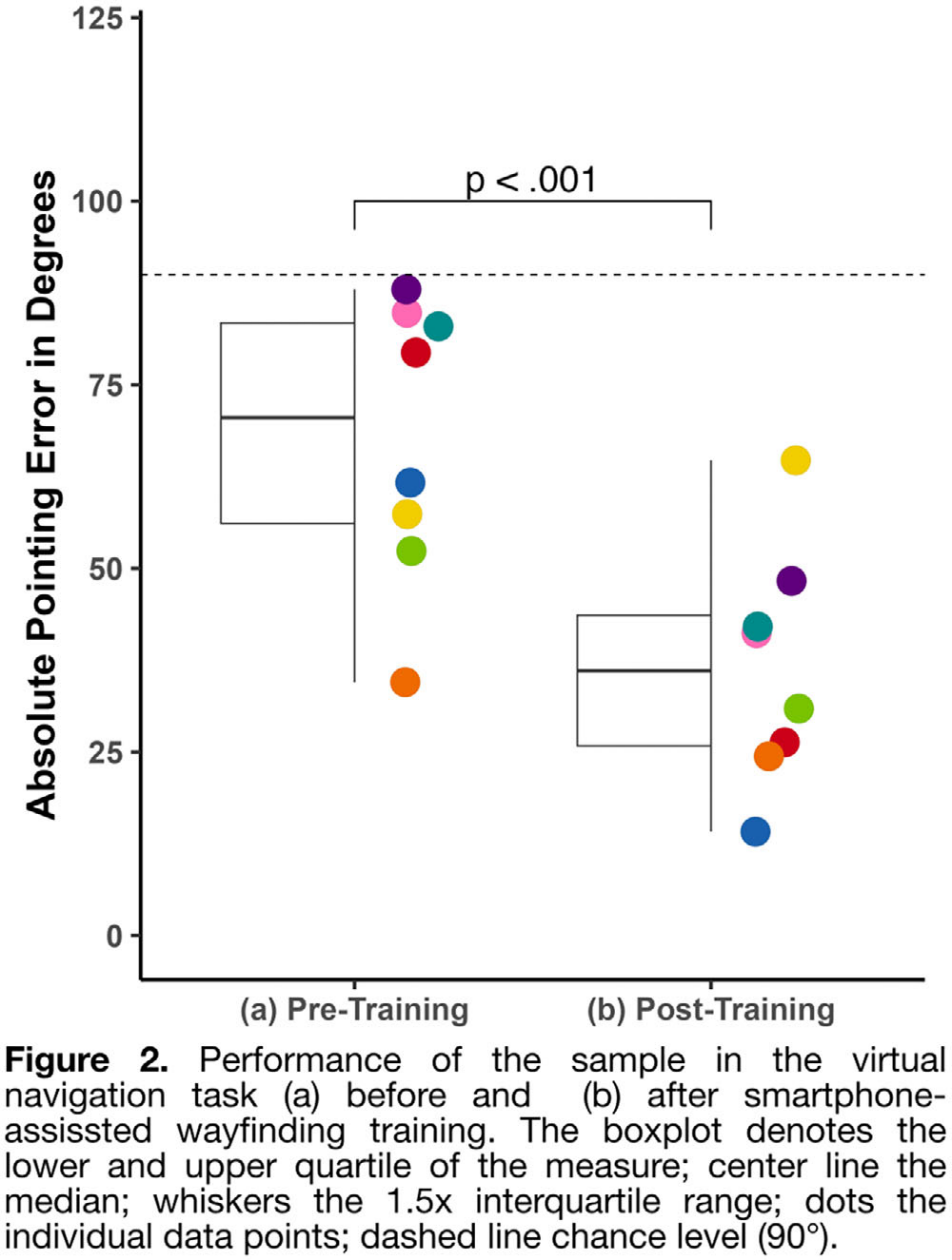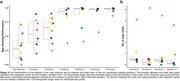# The impact of a smartphone‐assisted real‐world wayfinding training on spatial memory performance as measured in VR in older adults

**DOI:** 10.1002/alz.095264

**Published:** 2025-01-09

**Authors:** Jonas Marquardt, Esther Kuehn, Stefanie Schreiber, Anne Maass, Nadine Diersch

**Affiliations:** ^1^ German Center for Neurodegenerative Diseases (DZNE), Magdeburg Germany; ^2^ Hertie Institute for Clinical Brain Research, Tübingen Germany; ^3^ German Center for Neurodegenerative Diseases (DZNE), Tübingen Germany; ^4^ Institute for Cognitive Neurology and Dementia Research (IKND), Otto‐von‐Guericke University, Magdeburg Germany; ^5^ Department of Neurology, Otto‐von‐Guericke University, Magdeburg Germany; ^6^ Centre for Behavioural Brain Sciences (CBBS), Magdeburg, Sachsen‐Anhalt Germany; ^7^ Otto von Guericke University, Magdeburg Germany; ^8^ Center for Behavioral Brain Sciences (CBBS), Magdeburg Germany

## Abstract

**Background:**

Non‐pharmacological interventions that improve cognitive functioning in patients with Alzheimer’s disease will play a crucial role in the coming years to support their independence in daily living. Here, we investigated the potential of a novel smartphone‐assisted real‐world wayfinding training, tailored for older adults, to improve their spatial memory as one of the first cognitive functions affected by the disease. Spatial memory performance was assessed using a VR‐based navigation task and the Gardony map drawing analyzer.

**Method:**

To date, eight cognitively healthy older adults (65‐73 years) completed the wayfinding training on the medical campus area in Magdeburg, Germany, using our smartphone application “Explore” (Figure 1). In two egocentric sessions, participants had to walk repeatedly from a fixed start location to a target location and back. In four allocentric sessions, participants had to walk to several target locations consecutively by planning a route (four routes per session). All target locations were shown on a map in the app at the beginning of a track, which disappeared during walking but could be re‐opened. The app recorded GPS data and the number of map views. Potential changes in spatial memory were assessed by means of a pointing task in a virtual campus version before and after the training and by comparing the accuracy in the computerized map drawing after each session.

**Result:**

A linear mixed‐effect model analysis confirmed a significant training effect on pointing errors, while controlling for pre‐training familiarity, *p* < .001 (Figure 2). Map drawing accuracy improved over the course of the training, *p* < .001 (Figure 3a), while the number of map views during walking in allocentric sessions decreased, *p* = .021 (Figure 3b).

**Conclusion:**

We provide first evidence that a remotely administered real‐world wayfinding training might be able to improve spatial memory in older adults. As a next step, data will be collected in more participants with some of them assigned to a walking‐only control group. (f)MRI data, measured during the virtual pointing task, and biomarker status will be analyzed to investigate the mechanisms of the training effects and to determine the applicability as dementia intervention.